# Identification of Potential Common Pathogenic Mechanisms Underlying Osteoarthritis and Major Depressive Disorder Using Bioinformatics Analysis

**DOI:** 10.1002/iid3.70293

**Published:** 2025-11-25

**Authors:** Taiyuan Guan, Peixin Li, Sijian Su, Liang Ao, Xin Zhou

**Affiliations:** ^1^ Department of Orthopedics, The Affiliated Traditional Chinese Medicine Hospital Southwest Medical University Luzhou Sichuan China; ^2^ Luzhou Key Laboratory of Orthopedic Disorders Luzhou Sichuan China; ^3^ Institute of Integrated Chinese and Western Medicine Southwest Medical University Luzhou Sichuan China; ^4^ Southwest Medical University Luzhou Sichuan China

**Keywords:** comorbidity mechanism, CXCR6, GZMK, KLRG1, major depressive disorder, osteoarthritis

## Abstract

**Background:**

Patients with osteoarthritis (OA) exhibit an elevated risk for major depressive disorder (MDD), primarily due to chronic pain and associated disability. However, the shared molecular mechanisms underlying these conditions remain poorly understood.

**Methods:**

This study employs bioinformatics and systems biology approaches to identify common gene signatures and elucidate the shared pathogenesis of OA and MDD.

**Results:**

We identified 22 common differentially expressed genes between the two diseases, which were predominantly associated with the positive regulation of reactive oxygen species metabolic processes, immune and inflammatory responses, efferocytosis, the PI3K‐Akt signaling pathway, the TGF‐beta receptor signaling pathway, and immune system‐related pathways. Notably, CXCR6, GZMK, and KLRG1 were identified as key genes, showing positive correlations with CD8+ T cells and negative correlations with naïve CD4+ T cells and monocytes in both OA and MDD. Competitive endogenous RNA regulatory network analysis revealed that the KCNQ1OT1‐miR‐92a/miR‐132/miR‐19b/miR‐145‐CXCR6/GZMK/KLRG1 and XIST1‐miR‐92a/miR‐132/miR‐19b‐CXCR6/GZMK/KLRG1 regulatory axes may play critical roles in the pathogenesis of OA and MDD.

**Conclusion:**

These findings provide novel insights into the comorbidity mechanism of OA and MDD and may guide the development of individualized therapeutic strategies for patients with comorbid conditions.

## 1 Introduction

Osteoarthritis (OA), the most common chronic musculoskeletal disease, is characterized by persistent pain and disability, which significantly elevates the risk of comorbid depression and anxiety [1]. Research indicates that approximately 42% of OA patients experience major depressive disorder (MDD), a life‐threatening and debilitating psychiatric disorder worldwide [2]. Depression in OA patients is associated with greater weekly and momentary pain in complex ways. Moreover, comorbid depression has a higher likelihood of disability, lower therapeutic effect, and higher healthcare expenditures [3]. Risk factors of OA, such as elevated BMI, sex, neuronal impairment, and inflammatory factors, are also linked to depression [4, 5, 6]. The presence of symptomatic joints has been shown to correlate with higher self‐reported depression rates [7]. Additionally, OA is an inflammatory disease, and can induce the expression of the inflammatory factors to contribute to the development of depression. Both OA and MDD can exacerbate systemic inflammation and pro‐inflammatory cytokine production, leading to disease progression. Elevated levels of cytokines such as IL‐6, TNF, and CRP have been observed in MDD patients compared to healthy controls [8], suggesting a common inflammatory pathway that may underlie their comorbidity.

Recent genetic studies have underscored the genetic correlation between MDD and OA. Genome‐wide analyses of genetic variation identified several shared risk genes, including DRD2, FOXP2, NRXN1, and STRA6 [9]. Linkage disequilibrium score regression and mendelian randomization analysis revealed 29 shared genomic loci in MDD and OA, such as estrogen receptor 1 (ESR1), SRY‐Box Transcription Factor 5 (SOX5), and glutathione peroxidase 1 (GPX1) gene, which may provide important information for the treatment of patients with both diseases simultaneously [10], suggesting that concomitant depression in OA patients exacerbates disease management challenges and increases overall disease burden. Therefore, the interplay between OA and depression should be taken seriously. Despite those advancements, the precise molecular mechanisms underlying the OA–MDD association remain incompletely elucidated. Moreover, the lack of validated diagnostic markers for MDD in OA patients poses a significant challenge in clinical practice, as untreated depression can exacerbate OA symptoms, reduce treatment efficacy, and increase healthcare costs. Therefore, there is an urgent need to identify shared biomarkers and therapeutic targets to address both conditions concurrently.

In this study, we employ a bioinformatics approach to analyze high‐throughput gene expression data from OA and MDD patients, aiming to identify common differentially expressed genes (DEGs) and elucidate shared molecular pathways. By integrating functional enrichment analysis and protein–protein interaction (PPI) network analysis, we seek to uncover the molecular mechanisms underlying OA‐MDD comorbidity and provide a theoretical foundation for the development of diagnostic and therapeutic strategies for patients with comorbid OA and MDD, ultimately improving their quality of life and clinical outcomes.

## 2 Methods

### 2.1 Datasets Collection

Gene expression profiling data set of MDD and OA in this study was obtained from the Gene Expression Omnibus (GEO, http://www.ncbi.nlm.nih.gov/geo/) database. The GSE98793 data set contains 128 MDD peripheral whole blood samples and 64 healthy controls, applying the microarray [11]. The GSE48556 data set contains 106 OA samples and 33 healthy controls [12]. In addition, two commonly used datasets, GSE55235 for OA with synovial tissue and GSE201332 for MDD with peripheral whole blood, were used as validation [13, 14].

### 2.2 DEGs Identification

The R packages limma [15] was used to screen the DEGs with the criteria of |log2 Fold change| > 0.26 and adjusted *p*‐value < 0.05 between the OA and healthy group, MDD and normal sample, respectively. Then the common DEGs were obtained and displayed by Venn diagrams. In addition, the validation datasets (GSE55235 and GSE201332) were analyzed with the same screen criteria.

### 2.3 Functional Enrichment Analyses of the DEGs

Functional enrichment analyses, including Gene Ontology (GO) and Kyoto Encyclopedia of Genes and Genomes (KEGG) analyses of the DEGs, were performed using DAVID (https://david.ncifcrf.gov) [16], SRplot (https://www.bioinformatics.com.cn) [17], and Webgestalt2019 (https://www.webgestalt.org) [18]. The DEGs were uploaded to Metascape (https://metascape.org) [19] for gene function annotation.

### 2.4 PPI Network Analysis and Identification of the Hub Genes

PPI networks were constructed using the online database STRING (http://string‐db.org) [20] with combined scores > 0.4, and then visualized using Cytoscape (Version 3.9.1) [21]. In addition, the hub genes were identified by a plugin of Cytoscape, cytohubba (http://apps.cytoscape.org/apps/cytohubba) [22], which can explore important nodes and subnetworks in the PPI network. A co‐expression network of identified hub genes was constructed using the online GeneMANIA website (https://genemania.org) [23]. Moreover, the relationship between hub genes and disease was analyzed by the DisGeNET database [24], which contains information on genes and variants associated with human diseases.

### 2.5 Immune Cell Infiltration Correlation Analysis

To delineate the immune cell composition and the levels of immune cell infiltration in diseases, the CIBERSORT package in R was utilized. In addition, the correlation between the expression levels of hub genes and the degree of infiltration of various immune cell types was performed through Spearman's correlation analysis with Gene Set Enrichment Analysis (GSEA) [25].

### 2.6 lncRNA‐miRNA‐Gene Regulatory Network

TargetScan (https://www.targetscan.org/vert_80/) [26], miRDB (https://mirdb.org) [27], and miRWalk database (http://mirwalk.umm.uni‐heidelberg.de) [28] were used to establish the gene–miRNA interaction network. HMDD database (http://www.cuilab.cn/hmdd) [29] was used to obtain miRNA‐disease association data. The starbase database (https://rnasysu.com/encori/) [30] was used to predict the lncRNA–miRNA interaction. These networks were visualized using Cytoscape software, providing a comprehensive view of the potential regulatory relationships between the key gene and miRNAs.

## 3 Results

### 3.1 Identification of DEGs of OA and MDD

**Figure 1:**
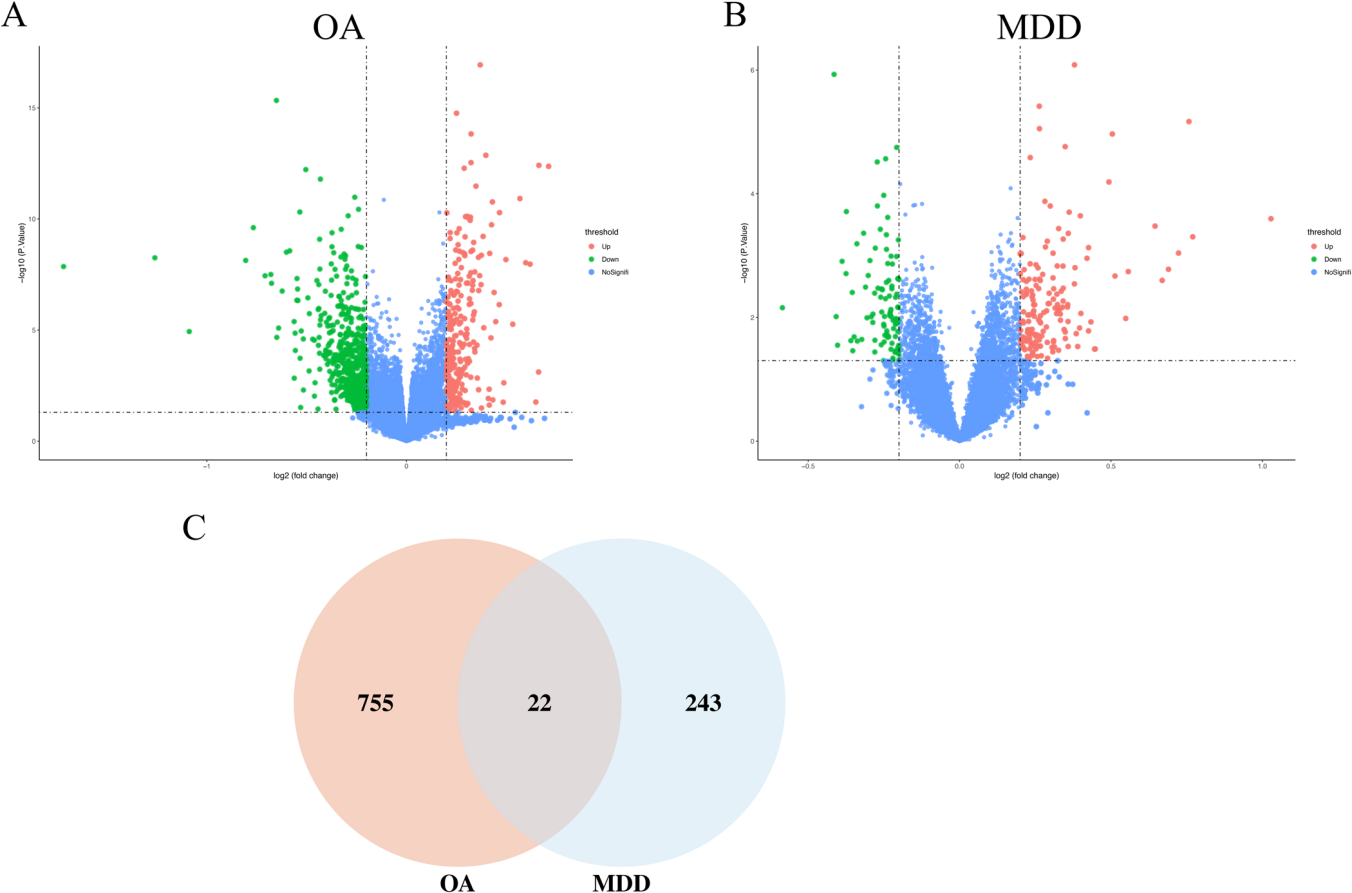
Identification of common differentially expressed genes (DEGs) between osteoarthritis (OA) and major depressive disorder (MDD) (A) Volcano plots of OA data. (B) Volcano plots of MDD data. (C) The Venn diagram showed the common DEGs between OA and MDD.

The gene expression profiling between OA patients and the control group was performed, and a total of 1115 upregulated and 1809 downregulated DEGs were identified and visualized by a volcano plot (Figure 1A). In addition, DEGs were identified between MDD patients and the healthy group and visualized by a volcano plot (Figure 1B). Then we obtained 22 common genes between both diseases with the Venn plot (Figure 1C).

### 3.2 Functional Enrichment Analysis and PPI Networks Construction of the Overlapping DEGs

**Figure 2:**
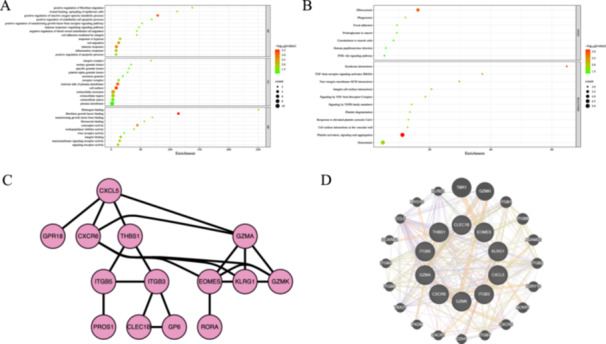
Functional enrichment analysis and PPI networks construction of the overlapped DEGs. (A) Gene Ontology annotations. (B) KEGG pathway enrichment data for the common DEGs. (C) The PPI network of DEGs was established with the String database. (D) The PPI network of DEGs was established with the GeneMANIA database.

To evaluate the function of the overlapping DEGs in the pathogenesis of OA and MDD, functional enrichment analysis was performed. GO enrichment analysis revealed that these overlapping DEGs were enriched in biological processes such as positive regulation of reactive oxygen species metabolic process, immune response, inflammatory response, response to hypoxia, and positive regulation of fibroblast migration in the biological process category. In the cellular component category, these genes were primarily located in the cell surface, the external side of the plasma membrane, and the extracellular exosome. Molecular function analysis indicated that these genes were mainly associated with fibroblast growth factor binding, coreceptor activity, fibrinogen binding, integrin binding, and transmembrane signaling receptor activity (Figure 2A). Moreover, pathway enrichment analysis demonstrates that the differential proteins were mainly involved in efferocytosis, PI3K‐Akt signaling pathway, TGF‐beta receptor signaling pathway, and immune system‐related pathway (Figure 2B). In addition, to construct PPI networks, the overlapping DEGs were entered into the String database and GeneMANIA database. The results were downloaded and visualized using Cytoscape (Figure 2C,D).

### 3.3 Hub Genes Regulatory Network and Immune Cell Infiltration

**Figure 3:**
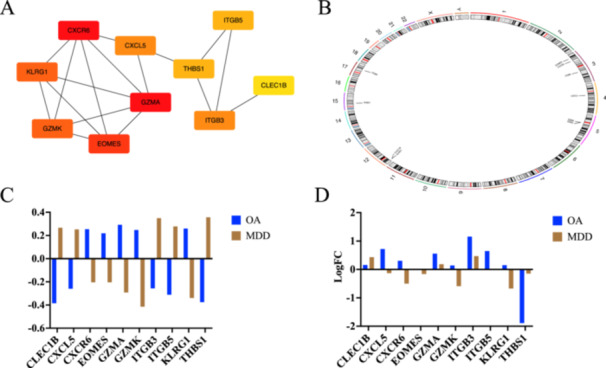
Identification and validation of hub genes in osteoarthritis (OA) and major depressive disorder (MDD). (A) The hub gene analysis of DEGs with Cytohubber. (B) Chromosome mapping of the hub genes. (C) The expression of the hub genes. (D) The expression levels of these hub genes were validated using the validated datasets (GSE55235 for OA and GSE201332 for MDD).

**Figure 4:**
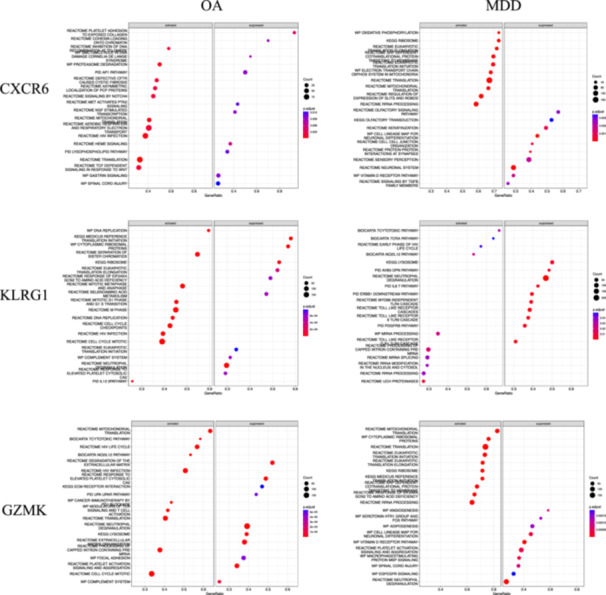
The function and enriched pathway of the identified hub genes CXCR6, GZMK, and KLRG1 in OA and MDD. CXCR6: GSEA of CXCR6 in OA and MDD. KLRG1: GSEA of KLRG1 in OA and MDD. GZMK: GSEA of GZMK in OA and MDD.

**Figure 5:**
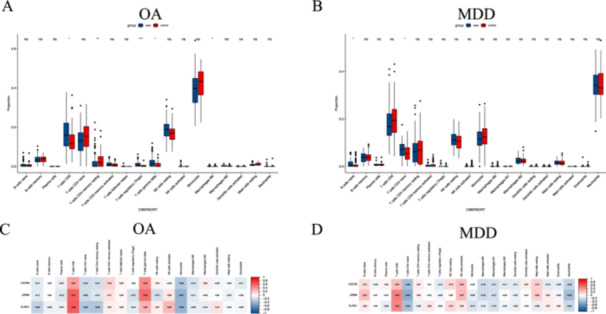
Immune cell infiltration analysis. (A) and (B) Violin diagram of the proportion of 22 types of immune cells of OA and MDD. (C) and (D) the difference infiltration of immune cells of the identified hub genes CXCR6, GZMK, and KLRG1 in OA and MDD.

CXCR6, KLRG1, GZMK, EOMES, GZMA, CXCL5, THBS1, ITGB5, ITGB3, and CLEC1B were identified as hub genes based on Cytoscape's plug‐in cytoHubba and the maximal clique centrality algorithm, and the chromosomal position analysis found that three genes were specifically located on chromosome 3 (Figure 3A,B). Interestingly, the expression levels of CXCR6, KLRG1, GZMK, EOMES, and GZMA were decreased in MDD patients, while they were upregulated in OA patients, and the expression levels of CXCL5, THBS1, ITGB5, ITGB3, and CLEC1B were increased in MDD patients, while they were downregulated in OA patients (Figure 3C). Moreover, the expression level of CXCR6, GZMK, and KLRG1 was consistent with the training datasets of both diseases (Figure 3D) by analyzing the validated datasets (GSE55235 for OA and GSE201332 for MDD), suggesting that CXCR6, GZMK, and KLRG1 may act as key genes related to OA and MDD. Furthermore, GSEA was performed to gain a more accurate understanding of the functional roles of the hub genes. For CXCR6, the enriched pathways were related to translation and mitochondrial translation in both diseases. For KLRG1, cell cycle, infection, and immune‐related pathways were enriched in both diseases. For GZMK, the enriched pathways included translation, mitochondrial translation, platelet activation signaling, and aggregation in both diseases (Figure 4). Additionally, the differences in the immune microenvironment between disease and healthy samples were analyzed with the CIBERSORT algorithm. We found that higher levels of CD8 T cells, T cells regulatory (Tregs), T cells gamma delta, and macrophages M0 were detected in OA patients compared to healthy, while the level of B cells naïve and T cells CD4 naïve was significantly increased in MDD patients compared to healthy (Figure 5A,B). Moreover, we analyzed the relationship between the key genes and immune cells, and found that CXCR6, GZMK, and KLRG1 were positively correlated with T cells CD8, and negatively correlated with T cells CD4 naïve and monocytes in both OA and MDD (Figure 5C,D).

### 3.4 Competitive Endogenous RNA (CeRNA) Regulatory Network Construction of the Key Genes

**Figure 6:**
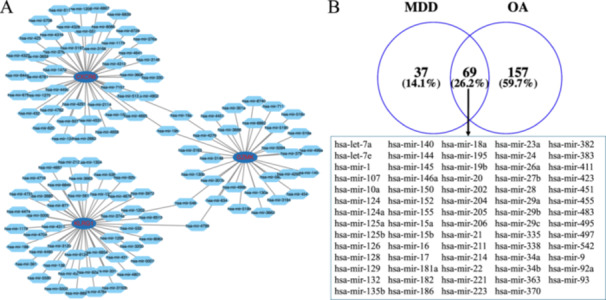
The interaction between miRNA and the key genes. (A) The predicted miRNAs are potentially associated with CXCR6, GZMK, and KLRG1. (B) The common miRNAs in both OA and MDD.

**Figure 7:**
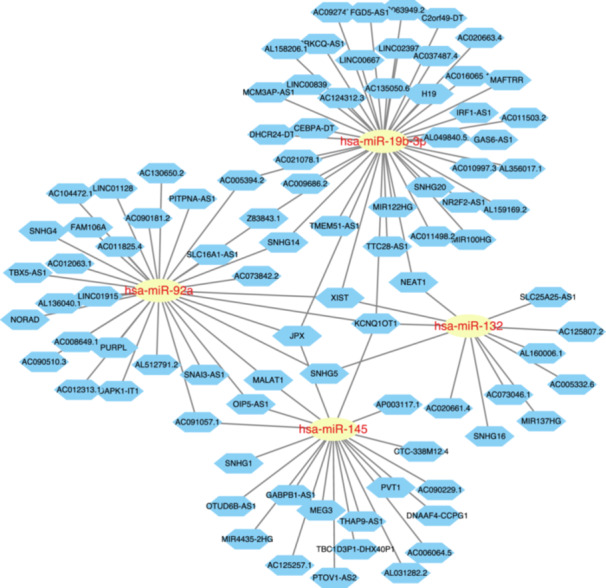
ceRNA regulatory network construction based on lncRNA‐miRNA, the key genes.

The TargetScan, miRDB, and miRWalk databases were utilized to predict miRNAs potentially associated with CXCR6, GZMK, and KLRG1 (Figure 6A). Additionally, based on the HMDD database, we obtained 69 miRNAs common to both diseases (Figure 6B). From these results, we found that hsa‐mir‐145, hsa‐mir‐19b, hsa‐mir‐124, hsa‐mir‐132, and hsa‐mir‐92a may target three key genes (CXCR6, GZMK, and KLRG1)and regulate the processes of both diseases. Furthermore, a ceRNA network was constructed using the starBase database to predict the interaction between lncRNAs and these five miRNAs (hsa‐mir‐145, hsa‐mir‐19b, hsa‐mir‐124, hsa‐mir‐132, and hsa‐mir‐92a). The analysis revealed that lncRNAs such as KCNQ1OT1, XIST, NEAT1, JPX, SNHG5, MALAT1, OIP‐AS1, and AC091057.1 competitively bind to at least two miRNAs (Figure 7). By integrating the regulatory relationships among lncRNA, miRNA, and mRNA, we propose that the KCNQ1OT1‐miR‐92a/miR‐132/miR‐19b/miR‐145‐CXCR6/GZMK/KLRG1, XIST1‐miR‐92a/miR‐132/miR‐19b‐CXCR6/GZMK/KLRG1 regulatory axis might represent critical pathways in the pathogenesis of OA and MDD.

## 4 Discussion

MDD is one of the most common comorbidities in OA, resulting in a higher prevalence and worse prognosis [31, 32]. Therefore, the effective diagnostic and intervention measures for depressive symptoms are important for patients with OA. Several biomarkers for OA or MDD have been identified [33, 34], but there are currently no markers for diagnosing MDD in patients with OA, and the interplay between OA, MDD, and biomarkers remains poorly understood. In this study, we performed bioinformatics analysis with GEO datasets to identify 22 overlapping DEGs, which were primarily enriched in pathways such as efferocytosis, PI3K‐Akt signaling pathway, TGF‐beta receptor signaling pathway, and immune system‐related pathway. The PI3K‐Akt signaling pathway is a crucial intracellular signaling cascade and is closely linked with the pathogenesis of OA and MDD. The PI3K‐Akt signaling pathway is associated with cartilage degradation, subchondral bone dysfunction, and inflammation, which are key features of OA [35, 36, 37]. In addition, the PI3K/Akt pathway has been found to be involved in neurotrophic factor signaling and can regulate glucose metabolism and energy homeostasis in the brain [38, 39]. Moreover, the PI3K/Akt pathway can regulate the activity of immune cells and the production of inflammatory mediators to participate in MDD progression [40, 41], suggesting that proteins involved in these processes may be potential targets for regulating OA and MDD. OA and MDD accelerate inflammatory response and tissue damage, leading to joint swelling, pain, and functional impairment of the central nervous system (CNS). Inflammation has been implicated in the PI3K/Akt pathway and TGF‐beta receptor signaling pathway [42, 43, 44, 45]. Taken together, we suggest that anti‐inflammatory treatments may potentially benefit both conditions.

Subsequently, we identified three key genes (CXCR6, GZMA, and KLRG1) that may be used for the diagnosis of the different periods of comorbid MDD with OA and found that the expression pattern of these three genes was different between OA and MDD, speculating that CXCR6, GZMA, and KLRG1 may present different roles during the different stages of disease development. ceRNA regulatory network construction revealed that ncRNA might affect the function of key genes, such as CXCR6, GZMK, and KLRG1, in the pathogenesis of OA and MDD. LncRNA KCNQ1OT1 and XIST were involved in inflammation and the pathogenesis of OA. Inhibition of lncRNA KCNQ1OT1 and XIST suppressed inflammatory cytokine expression [46, 47]. MiR‐145 was associated with MDD and targeted GZMK to regulate myocardial ischemia/reperfusion injury [48, 49]. miR‐132 has been proven to play a key role in the pathogenesis of depression and OA [50, 51].

Our result demonstrates the opposite expression pattern of CXCR6, GZMK, and KLRG1 in OA and MDD, suggesting that these genes may regulate different T cell subtypes to affect OA and MDD. The chemokine receptor CXCR6, which belongs to the G protein‐coupled receptor superfamily, binds to its ligand CXCL16 and is involved in cell‐to‐cell communication and the regulation of immune responses. It has emerged as a crucial molecule with diverse functions in a wide range of diseases. CXCR6/CXCL16 is highly expressed in the synovial tissue of rheumatoid arthritis (RA) patients, where it recruits T cells and monocytes to the inflamed joints, promoting the production of pro‐inflammatory cytokines that exacerbate joint inflammation and damage. CXCR6 deficiency increases pro‐inflammatory cytokine production in microglia and impairs the accumulation, tissue residency programming, and clonal expansion of brain PD‐1+CD8+ T cells [52]. Deficiency of CXCR6, a common chemokine receptor expressed by type II innate lymphoid cells (ILC2s), leads to a reduction in ILC2s, which are known to modulate neuroinflammation in the CNS [53, 54]. Although the direct interaction between CXCR6 and OA or MDD has not been well established, further investigation is required to elucidate its role in inflammatory responses under these conditions. Granzyme K (GZMK), a member of the granzyme family of proapoptotic serine proteases, plays important roles in cytotoxicity and pro‐inflammatory immune cytokine response associated with various diseases. GZMK has been implicated in the pathogenesis of autoimmune diseases, including RA and OA. Single‐cell RNA‐seq analysis has revealed a GZMK^+^ CD8 T cell subset in the synovial fluid and tissue that promotes inflammation by inducing the production of IL‐6, IL‐8, and CCL2 in RA. Furthermore, GZMK interacts with CCL5 to activate the ERK signaling pathway, thereby facilitating RA progression [55]. Proteome‐wide mendelian randomization analysis has identified GZMK as a core therapeutic target for knee OA, with genetic variants and colocalization evidence supporting its association with OA [56]. Killer cell lectin‐like receptor G1 (KLRG1) belongs to the C‐type lectin‐like receptor family and plays a crucial role in regulating cell‐mediated immunity within the immune system. KLRG1 can be expressed at the surface of T cells and natural killer cells, and can mediate T cell functions [57]. In addition, it has been shown that pathogenic PD‐1^+^ HLA‐DR^HIGH^ KLRG1^LOW^ T cells in synovial fluid may act as potential targets for future PD‐1 agonist therapies in inflammatory diseases [58]. KLRG1 is also induced in highly cytotoxic and proliferative effector CD8^+^ T cells and is associated with inflammatory signals [59]. Moreover, KLRG1, as the E‐cadherin ligand, interacts with E‐cadherin‐expressing DCs and macrophages to regulate PI3K/Akt, Rho GTPase, and NF‐κB signaling, which increases the production of pro‐inflammatory cytokines, such as TNF‐α and IL‐1β by macrophages and other immune cells in the joint [60, 61, 62, 63]. KLRG1 reduction resulted in a significant improvement in CD4(+) T cell proliferation [64], which is consistent with our results. It has been shown that the increased infiltration of CD8+ T cells and macrophages promoted inflammatory response in OA, and CD4+ T cells are associated with the development of MDD [65, 66]. In summary, based on our findings, we speculate that CXCR6, GZMK, and KLRG1 may play significant roles in inflammation that is associated with OA and MDD pressure, and will be potential biomarkers for OA and MDD comorbidity. However, our study still has some limitations. The impact of batch effects on the results requires an increase in sample size and data analysis, and the results should be verified using independent samples. In addition, the function and mechanism of key genes in the comorbidity process of OA and MDD are investigated using gene editing and single‐cell sequencing. Taken together, further investigation is required to provide insights into the inflammatory response in these conditions.

## 5 Conclusion

Based on bioinformatics analysis, we systematically identified three related candidate genes (CXCR6, GZMK, and KLRG1) that were positively correlated with T cells CD8, and negatively correlated with T cells CD4 naïve and monocytes in both OA and MDD. They were enriched in the inflammatory and PI3K‐Akt signaling pathway. Moreover, ceRNA regulatory network analysis found that KCNQ1OT1‐miR‐92a/miR‐132/miR‐19b/miR‐145‐CXCR6/GZMK/KLRG1, XIST‐miR‐92a/miR‐132/miR‐19b‐CXCR6/GZMK/KLRG1 regulatory axis may represent critical pathways in the pathogenesis of OA and MDD. Taken together, CXCR6, GZMK, and KLRG1could be used for clinical diagnosis and treatment for both diseases, and these findings may provide valuable insights for the comorbidity mechanisms of OA and MDD.

## Author Contributions

Taiyuan Guan designed this study, wrote this manuscript, and obtained funding. Peixin Li and Sijian Su conducted the data analysis. Liang Ao and Xin Zhou collected the data and revised the manuscript. All authors agreed on the final manuscript.

## Ethics Statement

The authors have nothing to report.

## Consent

The authors have nothing to report.

## Conflicts of Interest

The authors declare no conflicts of interest.

## Data Availability

The original contributions presented in the study are included in the article/supporting material.

## References

[1] B. Agustini , M. Lotfaliany , R. L. Woods , et al., “Patterns of Association Between Depressive Symptoms and Chronic Medical Morbidities in Older Adults,” Journal of the American Geriatrics Society 68, no. 8 (2020): 1834–1841, 10.1111/jgs.16468. 32402115 PMC7879564

[2] R. O. Akintayo , A. Yerima , H. B. Olaosebikan , C. Uhunmwangho , and A. A. Akpabio , “How Much Gloom Is in Groans? Depression and Its Determinants in Nigerian Patients With Knee Osteoarthritis: A Multi‐Center Cross‐Sectional Study,” Clinical Rheumatology 38 (2019): 1971–1978, 10.1007/s10067-019-04497-2. 30847688

[3] B. W. Smith and A. J. Zautra , “The Effects of Anxiety and Depression on Weekly Pain in Women With Arthritis,” Pain 138, no. 2 (2008): 354–361, 10.1016/j.pain.2008.01.008. 18289792 PMC2684788

[4] S. Zheng , L. Tu , F. Cicuttini , et al., “Depression in Patients With Knee Osteoarthritis: Risk Factors and Associations With Joint Symptoms,” BMC Musculoskeletal Disorders 22, no. 1 (2021): 40, 10.1186/s12891-020-03875-1. 33413273 PMC7791830

[5] J. Liu , H. Jin , D. K. Yon , et al., “Risk Factors for Depression in Patients With Knee Osteoarthritis: A Systematic Review and Meta‐Analysis,” Orthopedics 47, no. 5 (2024): e225–e232, 10.3928/01477447-20240821-10. 39208396

[6] J. J. McDougall , “Osteoarthritis Is a Neurological Disease – An Hypothesis,” Osteoarthritis and cartilage open 1, no. 1–2 (2019): 100005, 10.1016/j.ocarto.2019.100005. 36474723 PMC9718286

[7] R. Gandhi , M. G. Zywiel , N. N. Mahomed , and A. V. Perruccio , “Depression and the Overall Burden of Painful Joints: An Examination Among Individuals Undergoing Hip and Knee Replacement for Osteoarthritis,” Arthritis 2015 (2015): 327161, 10.1155/2015/327161. 25861476 PMC4377445

[8] S. A. Syed , E. Beurel , D. A. Loewenstein , et al., “Defective Inflammatory Pathways in Never‐Treated Depressed Patients Are Associated With Poor Treatment Response,” Neuron 99, no. 5 (2018): 914–924.e3, 10.1016/j.neuron.2018.08.001. 30146307 PMC6151182

[9] S. Barowsky , J. Y. Jung , N. Nesbit , et al., “Cross‐Disorder Genomics Data Analysis Elucidates a Shared Genetic Basis Between Major Depression and Osteoarthritis Pain,” Frontiers in Genetics 12 (2021): 687687, 10.3389/fgene.2021.687687. 34603368 PMC8481820

[10] F. Zhang , S. Rao , and A. Baranova , “Shared Genetic Liability Between Major Depressive Disorder and Osteoarthritis,” Bone & Joint Research 11, no. 1 (January 2022): 12–22, 10.1302/2046-3758.111.BJR-2021-0277.R1. 35023758 PMC8801173

[11] G. G. R. Leday , P. E. Vértes , S. Richardson , et al., “Replicable and Coupled Changes in Innate and Adaptive Immune Gene Expression in Two Case‐Control Studies of Blood Microarrays in Major Depressive Disorder,” Biological Psychiatry 83, no. 1 (2018): 70–80, 10.1016/j.biopsych.2017.01.021. 28688579 PMC5720346

[12] Y. F. M. Ramos , S. D. Bos , N. Lakenberg , et al., “Genes Expressed in Blood Link Osteoarthritis With Apoptotic Pathways,” Annals of the Rheumatic Diseases 73, no. 10 (2014): 1844–1853, 10.1136/annrheumdis-2013-203405. 23864235

[13] D. Woetzel , R. Huber , P. Kupfer , et al., “Identification of Rheumatoid Arthritis and Osteoarthritis Patients by Transcriptome‐Based Rule Set Generation,” Arthritis Research & Therapy 16, no. 2 (2014): R84, 10.1186/ar4526. 24690414 PMC4060460

[14] J. Xiu , J. Li , Z. Liu , et al., “Elevated BICD2 DNA Methylation in Blood of Major Depressive Disorder Patients and Reduction of Depressive‐Like Behaviors in Hippocampal Bicd2‐Knockdown Mice,” Proceedings of the National Academy of Sciences 119, no. 30 (July 2022): e2201967119, 10.1073/pnas.2201967119. PMC9335189 35858435

[15] M. E. Ritchie , B. Phipson , D. Wu , et al., “Limma Powers Differential Expression Analyses for RNA‐Sequencing and Microarray Studies,” Nucleic Acids Research 43, no. 7 (2015): e47, 10.1093/nar/gkv007. 25605792 PMC4402510

[16] G. Dennis , B. T. Sherman , D. A. Hosack , et al., “DAVID: Database for Annotation, Visualization, and Integrated Discovery,” Genome Biology 4, no. 5 (2003): P3.12734009

[17] D. Tang , M. Chen , X. Huang , et al., “SRplot: A Free Online Platform for Data Visualization and Graphing,” PLoS One 18, no. 11 (2023): e0294236, 10.1371/journal.pone.0294236. 37943830 PMC10635526

[18] Y. Liao , J. Wang , E. J. Jaehnig , Z. Shi , and B. Zhang , “WebGestalt 2019: Gene Set Analysis Toolkit With Revamped UIs and APIs,” Nucleic Acids Research 47, no. W1 (2019): W199–W205, 10.1093/nar/gkz401. 31114916 PMC6602449

[19] Y. Zhou , B. Zhou , L. Pache , et al., “Metascape Provides a Biologist‐Oriented Resource for the Analysis of Systems‐Level Datasets,” Nature Communications 10, no. 1 (2019): 1523, 10.1038/s41467-019-09234-6. PMC6447622 30944313

[20] D. Szklarczyk , R. Kirsch , M. Koutrouli , et al., “The STRING Database in 2023: Protein‐Protein Association Networks and Functional Enrichment Analyses for Any Sequenced Genome of Interest,” Nucleic Acids Research 51, no. D1 (2023): D638–D646, 10.1093/nar/gkac1000. 36370105 PMC9825434

[21] P. Shannon , A. Markiel , O. Ozier , et al., “Cytoscape: A Software Environment for Integrated Models of Biomolecular Interaction Networks,” Genome Research 13, no. 11 (2003): 2498–2504, 10.1101/gr.1239303. 14597658 PMC403769

[22] C. H. Chin , S. H. Chen , H. H. Wu , C. W. Ho , M. T. Ko , and C. Y. Lin , “cytoHubba: Identifying Hub Objects and Sub‐Networks From Complex Interactome,” supplement, BMC Systems Biology 8, no. Suppl 4 (2014): S11, 10.1186/1752-0509-8-S4-S11. 25521941 PMC4290687

[23] M. Franz , H. Rodriguez , C. Lopes , et al., “GeneMANIA Update 2018,” Nucleic Acids Research 46, no. W1 (2018): W60–W64, 10.1093/nar/gky311. 29912392 PMC6030815

[24] J. Piñero , À. Bravo , N. Queralt‐Rosinach , et al., “DisGeNET: A Comprehensive Platform Integrating Information on Human Disease‐Associated Genes and Variants,” Nucleic Acids Research 45, no. D1 (2017): D833–D839, 10.1093/nar/gkw943. 27924018 PMC5210640

[25] A. M. Newman , C. L. Liu , M. R. Green , et al., “Robust Enumeration of Cell Subsets From Tissue Expression Profiles,” Nature Methods 12, no. 5 (May 2015): 453–457, 10.1038/nmeth.3337. 25822800 PMC4739640

[26] S. E. McGeary , K. S. Lin , C. Y. Shi , et al., “The Biochemical Basis of MicroRNA Targeting Efficacy,” Science 366, no. 6472 (2019): eaav1741, 10.1126/science.aav1741. 31806698 PMC7051167

[27] Y. Chen and X. Wang , “miRDB: An Online Database for Prediction of Functional MicroRNA Targets,” Nucleic Acids Research 48, no. D1 (2020): D127–D131, 10.1093/nar/gkz757. 31504780 PMC6943051

[28] C. Sticht , C. De La Torre , A. Parveen , and N. Gretz , “miRWalk: An Online Resource for Prediction of MicroRNA Binding Sites,” PLoS One 13, no. 10 (2018): e0206239, 10.1371/journal.pone.0206239. 30335862 PMC6193719

[29] C. Cui , B. Zhong , R. Fan , and Q. Cui , “HMDD v4.0: A Database for Experimentally Supported Human MicroRNA‐Disease Associations,” Nucleic Acids Research 52, no. D1 (2024): D1327–D1332, 10.1093/nar/gkad717. 37650649 PMC10767894

[30] J. H. Li , S. Liu , H. Zhou , L. H. Qu , and J. H. Yang , “starBase v2.0: Decoding miRNA‐ceRNA, miRNA‐ncRNA and Protein‐RNA Interaction Networks From Large‐Scale CLIP‐Seq Data,” Nucleic Acids Research 42 (2014): D92–D97, 10.1093/nar/gkt1248. 24297251 PMC3964941

[31] K. B. Diamond , A. M. Gordon , B. K. Sheth , A. A. Romeo , and J. Choueka , “How Does Depressive Disorder Impact Outcomes in Patients With Glenohumeral Osteoarthritis Undergoing Primary Reverse Shoulder Arthroplasty?,” Journal of Shoulder and Elbow Surgery 32, no. 9 (2023): 1886–1892, 10.1016/j.jse.2023.03.013. 37044306

[32] B. Li , Z. Yang , Y. Li , J. Zhang , C. Li , and N. Lv , “Exploration Beyond Osteoarthritis: The Association and Mechanism of Its Related Comorbidities,” Frontiers in Endocrinology 15 (2024): 1352671, 10.3389/fendo.2024.1352671. 38779455 PMC11110169

[33] A. Mobasheri , C. S. Thudium , A. C. Bay‐Jensen , et al., “Biomarkers for Osteoarthritis: Current Status and Future Prospects,” Best Practice & Research Clinical Rheumatology 37, no. 2 (2023): 101852, 10.1016/j.berh.2023.101852. 37620236

[34] S. G. Kang and S. E. Cho , “Neuroimaging Biomarkers for Predicting Treatment Response and Recurrence of Major Depressive Disorder,” International Journal of Molecular Sciences 21, no. 6 (2020): 2148, 10.3390/ijms21062148. 32245086 PMC7139562

[35] L. Jiang , X. Zhou , K. Xu , et al., “miR‐7/EGFR/MEGF9 Axis Regulates Cartilage Degradation in Osteoarthritis via PI3K/AKT/mTOR Signaling Pathway,” Bioengineered 12, no. 1 (2021): 8622–8634, 10.1080/21655979.2021.1988362. 34629037 PMC8806962

[36] H. Li , S. Xie , Y. Qi , H. Li , R. Zhang , and Y. Lian , “TNF‐α Increases the Expression of Inflammatory Factors in Synovial Fibroblasts by Inhibiting the PI3K/AKT Pathway in a Rat Model of Monosodium Iodoacetate‐Induced Osteoarthritis,” Experimental and Therapeutic Medicine 16, no. 6 (2018): 4737–4744, 10.3892/etm.2018.6770. 30542428 PMC6257214

[37] K. Sun , J. Luo , J. Guo , X. Yao , X. Jing , and F. Guo , “The PI3K/AKT/mTOR Signaling Pathway in Osteoarthritis: A Narrative Review,” Osteoarthritis and Cartilage 28, no. 4 (2020): 400–409, 10.1016/j.joca.2020.02.027. 32081707

[38] S. N. Rai , H. Dilnashin , H. Birla , et al., “The Role of PI3K/Akt and ERK in Neurodegenerative Disorders,” Neurotoxicity Research 35, no. 3 (2019): 775–795, 10.1007/s12640-019-0003-y. 30707354

[39] Y. J. Lu , C. Shao , S. Zhang , W. Shi , L. Li , and J. J. Zhao , “Electroacupuncture Regulates Glucose Metabolism Disorder Through PI3K/Akt/GSK3β Signaling Pathway in Rats With Depression,” Zhen ci yan jiu = Acupuncture Research 48, no. 3 (2023): 247–252, 10.13702/j.1000-0607.20211353. 36951076

[40] R. J. Ni , T. H. Gao , Y. Y. Wang , et al., “Chronic Lithium Treatment Ameliorates Ketamine‐Induced Mania‐Like Behavior via the PI3K‐AKT Signaling Pathway,” Zoological Research 43, no. 6 (November 2022): 989–1004, 10.24272/j.issn.2095-8137.2022.278. 36257830 PMC9700503

[41] N. Guo , X. Wang , M. Xu , J. Bai , H. Yu , and Z. Le Zhang , “PI3K/AKT Signaling Pathway: Molecular Mechanisms and Therapeutic Potential in Depression,” Pharmacological Research 206 (2024): 107300, 10.1016/j.phrs.2024.107300. 38992850

[42] S. Liu , Z. Deng , K. Chen , et al., “Cartilage Tissue Engineering: From Proinflammatory and Anti‐Inflammatory Cytokines to Osteoarthritis Treatments (Review),” Molecular Medicine Reports 25, no. 3 (2022): 99, 10.3892/mmr.2022.12615. 35088882 PMC8809050

[43] Y. Li , H. Wang , J. Zhou , and C. Wang , “Research Progress on the Correlation Between Transforming Growth Factor‐β Level and Symptoms of Depression,” Zhejiang da xue xue bao. Yi xue ban = Journal of Zhejiang University. Medical Sciences 52, no. 5 (2023): 646–652, 10.3724/zdxbyxb-2023-0269. 37916311 PMC10630060

[44] S. Mihailova , E. Ivanova‐Genova , T. Lukanov , V. Stoyanova , V. Milanova , and E. Naumova , “A Study of TNF‐α, TGF‐β, IL‐10, IL‐6, and IFN‐γ Gene Polymorphisms in Patients With Depression,” Journal of Neuroimmunology 293 (2016): 123–128, 10.1016/j.jneuroim.2016.03.005. 27049572

[45] M. H. Davami , R. Baharlou , A. Ahmadi Vasmehjani , et al., “Elevated IL‐17 and TGF‐β Serum Levels: A Positive Correlation Between T‐Helper 17 Cell‐Related Pro‐Inflammatory Responses With Major Depressive Disorder,” Basic and Clinical Neuroscience 7, no. 2 (2016): 137–142, 10.15412/J.BCN.03070207. 27303608 PMC4892318

[46] D. Aili , T. Wu , Y. Gu , Z. Chen , and W. Wang , “Knockdown of Long Non‐Coding RNA KCNQ1OT1 Suppresses the Progression of Osteoarthritis by Mediating the miR‐211‐5p/TCF4 Axis In Vitro,” Experimental and Therapeutic Medicine 21, no. 5 (2021): 455, 10.3892/etm.2021.9886. 33747189 PMC7967809

[47] W. Sun , M. Ma , H. Yu , and H. Yu , “Inhibition of lncRNA X Inactivate‐Specific Transcript Ameliorates Inflammatory Pain by Suppressing Satellite Glial Cell Activation and Inflammation by Acting as a Sponge of miR‐146a to Inhibit Nav 1.7,” Journal of Cellular Biochemistry 119, no. 12 (2018): 9888–9898, 10.1002/jcb.27310. 30129228

[48] Y. Y. Hung , C. K. Chou , Y. C. Yang , H. C. Fu , E. W. Loh , and H. Y. Kang , “Exosomal let‐7e, miR‐21‐5p, miR‐145, miR‐146a and miR‐155 in Predicting Antidepressants Response in Patients With Major Depressive Disorder,” Biomedicines 9, no. 10 (2021): 1428, 10.3390/biomedicines9101428. 34680545 PMC8533438

[49] Z. Qi , S. Li , Y. Su , et al., “Role of microRNA‐145 in Protection Against Myocardial Ischemia/Reperfusion Injury in Mice by Regulating Expression of GZMK With the Treatment of Sevoflurane,” Journal of Cellular Physiology 234, no. 9 (2019): 16526–16539, 10.1002/jcp.28323. 30873621

[50] S. Qi , X. Yang , L. Zhao , et al., “MicroRNA132 Associated Multimodal Neuroimaging Patterns in Unmedicated Major Depressive Disorder,” Brain 141, no. 3 (2018): 916–926, 10.1093/brain/awx366. 29408968 PMC5837315

[51] W. Zhang , C. Hu , C. Zhang , C. Luo , B. Zhong , and X. Yu , “MiRNA‐132 Regulates the Development of Osteoarthritis in Correlation With the Modulation of PTEN/PI3K/AKT Signaling,” BMC Geriatrics 21, no. 1 (2021): 175, 10.1186/s12877-021-02046-8. 33691628 PMC7945330

[52] W. Su , J. Saravia , I. Risch , et al., “CXCR6 Orchestrates Brain CD8(+) T Cell Residency and Limits Mouse Alzheimer's Disease Pathology,” Nature Immunology 24, no. 10 (October 2023): 1735–1747, 10.1038/s41590-023-01604-z. 37679549 PMC11102766

[53] S. Meunier , S. Chea , D. Garrido , et al., “Maintenance of Type 2 Response by CXCR6‐Deficient ILC2 in Papain‐Induced Lung Inflammation,” International Journal of Molecular Sciences 20, no. 21 (2019): 5493, 10.3390/ijms20215493. 31690060 PMC6862482

[54] S. S. H. Yeung , Y. S. Ho , and R. C. C. Chang , “The Role of Meningeal Populations of Type II Innate Lymphoid Cells in Modulating Neuroinflammation in Neurodegenerative Diseases,” Experimental & Molecular Medicine 53, no. 9 (2021): 1251–1267, 10.1038/s12276-021-00660-5. 34489558 PMC8492689

[55] F. Zhang , K. Wei , K. Slowikowski , et al., “Defining Inflammatory Cell States in Rheumatoid Arthritis Joint Synovial Tissues by Integrating Single‐Cell Transcriptomics and Mass Cytometry,” Nature Immunology 20, no. 7 (2019): 928–942, 10.1038/s41590-019-0378-1. 31061532 PMC6602051

[56] M. Zou and Z. Shao , “Proteome‐Wide Mendelian Randomization and Colocalization Analysis Identify Therapeutic Targets for Knee and Hip Osteoarthritis,” Biomolecules 14, no. 3 (2024): 355, 10.3390/biom14030355. 38540773 PMC10967895

[57] H. Kared , S. Martelli , T. P. Ng , S. L. F. Pender , and A. Larbi , “CD57 in Human Natural Killer Cells and T‐Lymphocytes,” Cancer Immunology, Immunotherapy 65, no. 4 (2016): 441–452, 10.1007/s00262-016-1803-z. 26850637 PMC11029668

[58] J. Straube , S. Bukhari , S. Lerrer , et al., “PD‐1 Signaling Uncovers a Pathogenic Subset of T Cells in Inflammatory Arthritis,” Arthritis Research & Therapy 26, no. 1 (2024): 32, 10.1186/s13075-023-03259-5. 38254179 PMC10801937

[59] D. Herndler‐Brandstetter , H. Ishigame , R. Shinnakasu , et al., “KLRG1(+) Effector CD8(+) T Cells Lose KLRG1, Differentiate Into All Memory T Cell Lineages, and Convey Enhanced Protective Immunity,” Immunity 48, no. 4 (2018): 716–729.e8, 10.1016/j.immuni.2018.03.015. 29625895 PMC6465538

[60] J. Van den Bossche , B. Malissen , A. Mantovani , P. De Baetselier , and J. A. Van Ginderachter , “Regulation and Function of the E‐Cadherin/Catenin Complex in Cells of the Monocyte‐Macrophage Lineage and DCs,” Blood 119, no. 7 (2012): 1623–1633, 10.1182/blood-2011-10-384289. 22174153

[61] K. Sun , J. Luo , J. Guo , X. Yao , X. Jing , and F. Guo , “The PI3K/AKT/mTOR Signaling Pathway in Osteoarthritis: A Narrative Review,” Osteoarthritis and Cartilage 28, no. 4 (2020): 400–409, 10.1016/j.joca.2020.02.027. 32081707

[62] H. Zhang , D. Cai , and X. Bai , “Macrophages Regulate the Progression of Osteoarthritis,” Osteoarthritis and Cartilage 28, no. 5 (2020): 555–561, 10.1016/j.joca.2020.01.007. 31982565

[63] E. Malmhäll‐Bah , K. M. E. Andersson , M. C. Erlandsson , et al., “Rho‐GTPase Dependent Leukocyte Interaction Generates Pro‐Inflammatory Thymic Tregs and Causes Arthritis,” Journal of Autoimmunity 130 (2022): 102843, 10.1016/j.jaut.2022.102843. 35643017

[64] Y. Yang , W. Han , X. Zhang , et al., “Depression‐Related Innate Immune Genes and Pan‐Cancer Gene Analysis and Validation,” Frontiers in Genetics 15 (2025): 1521238, 10.3389/fgene.2024.1521238. 39867577 PMC11757255

[65] Y. S. Li , W. Luo , S. A. Zhu , and G. H. Lei , “T Cells in Osteoarthritis: Alterations and Beyond,” Frontiers in Immunology 8 (2017): 356, 10.3389/fimmu.2017.00356. 28424692 PMC5371609

[66] W. Shi , S. Zhang , Y. Lu , Y. Wang , J. Zhao , and L. Li , “T Cell Responses in Depressed Mice Induced by Chronic Unpredictable Mild Stress,” Journal of Affective Disorders 296 (2022): 150–156, 10.1016/j.jad.2021.09.064. 34601302

